# Treatment of Dysphagia in Parkinson’s Disease: A Systematic Review

**DOI:** 10.3390/ijerph17114104

**Published:** 2020-06-09

**Authors:** Remedios López-Liria, Jennifer Parra-Egeda, Francisco A. Vega-Ramírez, José Manuel Aguilar-Parra, Rubén Trigueros-Ramos, María José Morales-Gázquez, Patricia Rocamora-Pérez

**Affiliations:** 1 Health Research Centre, Department of Nursing, Physiotherapy and Medicine, University of Almería, Carretera del Sacramento s/n, La Cañada de San Urbano, 04120 Almería, Spain; rocamora@ual.es; 2 Poniente Hospital, Ctra. de Almerimar, s/n, El Ejido, 04700 Almería, Spain; jpe605@ilumine.ual.es; 3 Distrito Sanitario Poniente, Jesús de Perceval, 22. El Ejido, 04700 Almería, Spain; francisco.vega.ramirez.sspa@juntadeandalucia.es; 4 Department of Psychology, University of Almería, Carretera del Sacramento s/n, La Cañada de San Urbano, 04120 Almería, Spain; 5 Department of Nursing, University of Las Palmas de Gran Canaria (ULPGC), Juan de Quesada, 30, 35001 Las Palmas de Gran Canaria, Spain; mariajose.morales@ulpgc.es

**Keywords:** dysphagia, Parkinson, treatment, evaluation, quality of life, deglutition, rehabilitation, therapy

## Abstract

The incidence of oropharyngeal dysphagia in Parkinson’s disease (PD) is very high. It is necessary to search for effective therapies that could prevent pneumonia. Previous results should be interpreted cautiously as there is a lack of evidence to support the use of compensatory or rehabilitative approaches to dysphagia. We reviewed the scientific literature to describe the treatments of dysphagia in PD. A systematic review was performed in PubMed, Scopus, Elsevier, and Medline according to PRISMA standards in 2018. The articles that did not mention dysphagia secondary to PD or used surgical treatment were excluded. Eleven articles met the criteria with information from 402 patients. The review relates to different protocols, such as training in expiratory muscle strength, postural techniques, oral motor exercises, video-assisted swallowing therapy, surface electrical stimulation, thermal stimulation, touch, compensatory interventions, training regime for swallowing, neuromuscular electrical stimulation, Lee Silverman voice treatment, swallow maneuver, airway protection, and postural compensation maneuvers. This review identifies the rationing interventions in each trial, if they are efficient and equitable. Several rehabilitative therapies have been successful. An improvement was seen in the degenerative function (coordination, speed, and volume), quality of life, and social relationships of people with PD. Further investigations concerning the clinical applicability of these therapies based on well-designed randomized controlled studies are needed. Larger patient populations need to be recruited to evaluate the effectiveness, long-term effects, and new treatment techniques.

## 1. Introductions

Parkinson’s disease (PD) is a progressive and chronic neurodegenerative disorder, characterized by a deep and selective loss of dopaminergic neurons, with clinical manifestations including motor and nonmotor abnormalities [1,2]. PD is the second most common neurodegenerative disorder [3,4], and the average incidence rate estimated in developed countries is 14 cases per 100,000 people per year. If only subjects with 65 years or more age are considered, this rate increases to 160 cases per 100,000 persons per year. Mortality studies have shown the doubling of mortality rate in patients with PD compared with the general population. Given these epidemiological data, we can consider PD as a pathological disease with an enormous impact, which is expected to increase in the coming years, making it necessary to search for new therapies for PD [4]. In terms of prevalence, by taking into account all types of studies, in industrialized countries, the prevalence ranges from 0.3% to 1% in subjects with more than 60 years of age and reaches 3% in patients over 80 years of age. The number of studies on the prevalence, incidence, and mortality of PD has increased in recent years. Overall, prevalence and incidence vary widely across the world. The prevalence is higher in Europe and the United States than in other countries and distributed relatively evenly with a minimal range. In addition, PD increases mortality [5].

Among the leading causes of death in PD is pneumonia [6,7,8]. Langmore et al. [8] found that dysphagia is an important risk factor for the development of aspiration pneumonia when combined with other factors (such as feeding, functional, medical, or dental status; gastroesophageal reflux; colonization of the oropharynx with bacterial pathogens). Many of these are applicable to patients with PD. In addition, up to 80% of all patients will suffer from oropharyngeal dysphagia during the early stages of the disease. In the advanced stages of PD, the incidence of dysphagia may increase by up to 95% [9,10]. Treatment of dysphagia could, therefore, be one of the cornerstones for preventing pneumonia in PD patients [11,12].

Sensorimotor deficits affecting voice and swallowing ability can have a devastating impact on the quality of life of PD patients [13]. Former studies on the effects of speech and swallowing therapy in PD and physical and occupational therapy have demonstrated little or uncertain effects [12]. Nagaya et al. [10] evaluated the effect of swallowing training on dysphagia in PD without a control group and without an adequate baseline; therefore, its results should be interpreted cautiously. Sharkawi et al. [14] evaluated the effects of the Lee Silverman voice treatment (LVST) and found a 51% reduction in general swallowing disorders. A systematic review in 2010 revealed 19 publications that included targeted training for voice, and only one publication included targeted training for swallowing [13]. It is necessary to search for effective therapies that are useful in preventing pneumonia. The earlier results are not adequate for proper interpretation due to the lack of evidence to support the use of either compensatory or rehabilitative approaches in dysphagia. Our purpose was to organize and describe the benefits of different therapies in dysphagia associated with Parkinson’s disease in the last 10 years. Further research should be based on randomized controlled trials to determine the effectiveness and the clinical applicability of different therapies for dysphagia [11].

Dysphagia is a routine problem faced by health workers and family members. Dietary change and the use of thickeners are usual treatments, but they are also considerably expensive [15,16]. This study is justified because it is necessary to deepen research to identify the rationing instruments, which, if efficient and equitable, offer greater possibilities to improve health interventions in a population affected by neurological diseases that demand care processes and procedures that contribute to the quality of life [17,18]. In PD, ever more patients are dying from aspiration pneumonia [8]. Moreover, the quality of life is often diminished because PD does not allow patients to lead an active social life.

This study reviewed the scientific literature regarding the different techniques used to manage dysphagia in patients with PD.

The next section presents detailed information on the method employed in this review, which was based on the PRISMA guidelines [19]. All types of interventions and outcomes mentioned in the studies were included. This paper has intended to provide an overview of what is known about the treatments for dysphagia in PD, describing concise and precise updates on the progress made so far.

## 2. Methods

We performed a systematic review by following the PRISMA guidelines [19]. The articles were selected from the databases PubMed, Medline, Elsevier, and Scopus in 2018 by using the following descriptors in English: “Parkinson disease,” “disease of Parkinson,” “Parkinson disorders,” “deglutition disorders,” “dysphagia,” “oral care,” “mouth rehabilitation,” “rehabilitation,” “physical therapy specialty,” “physiotherapy,” “physiotherapy modalities,” and “physiotherapy treatment,”. In Spanish: “Parkinson,” “disfagia,” and “fisioterapia.”

The inclusion criteria were articles (clinical trials, pilot studies, and descriptive studies) that included a rehabilitation treatment or physiotherapy technique for patients with PD with dysphagia and should be published in the last ten years (from 2008 inclusive). Meta-analysis, systematic reviews, all articles that did not mention dysphagia secondary to PD or those that mentioned the application of surgical treatment were excluded.

Risk of bias assessment: the clinical trials were evaluated using the Jadad scale [20], a questionnaire that offers a score of 0 to 5, where the higher the score is, the better the methodological quality of the article. This scale includes five questions regarding randomization and the sequence; masking (as double-blind) and the method; and description of losses and exclusions (follow-up), with the answers ‘Yes’ (1 point) and ‘No’ (0 point). A clinical trial having a score of 5 is considered rigorous; a clinical trial having a score equal to or below 2 points is considered low-quality.

## 3. Results

**Figure 1:**
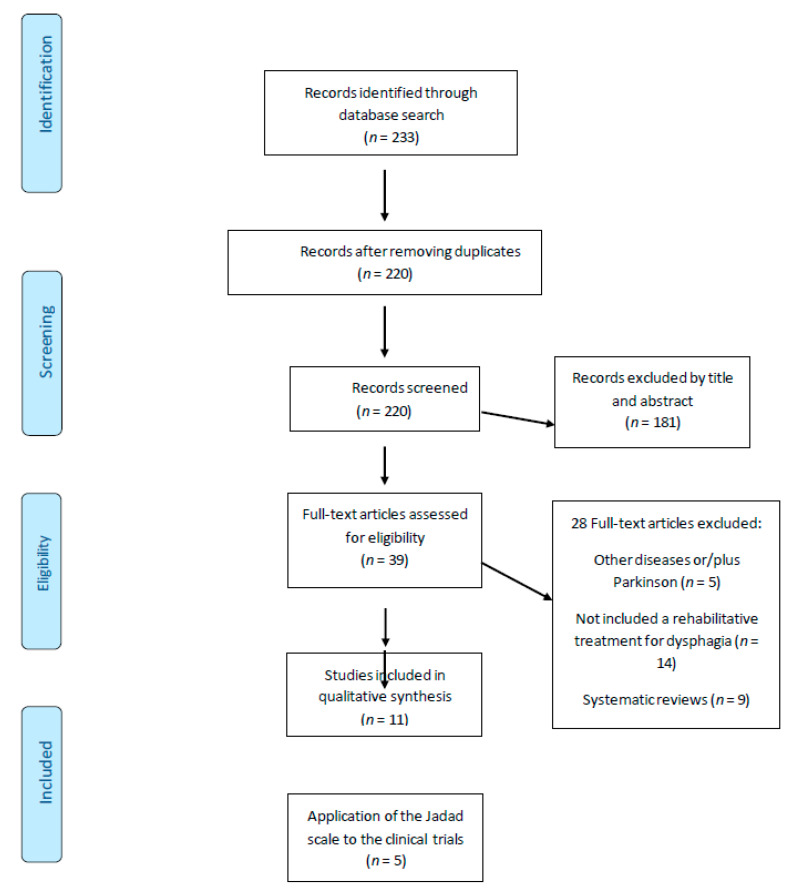
The selected articles from the databases.

The search strategy used to identify appropriate literature for this review within the databases is shown in Table 1. Figure 1 shows how articles were selected according to the study’s objective and the inclusion/exclusion criteria.

With this search strategy, 233 studies were found and reviewed according to the title and abstract by two independent evaluators (JPE and RLL) using the Rayyan QCRI program (https://rayyan.qcri.org), to include only those that met the eligibility criteria of the study. We removed 13 duplicate studies and excluded 181 by title and content of the abstract. Disagreements were settled by consensus and, if needed, by a third reviewer. After an initial screening of the studies that were considered potentially relevant (39 articles), a full-text reading was carried out, paying special attention to the study design, the intervention (population, treatment type, number of sessions and duration), and other factors. Five full-text articles were excluded as they did not mention dysphagia secondary to PD; 14 articles did not include rehabilitative treatment, while nine were systematic reviews. Finally, eleven articles met this study’s objective and inclusion criteria.

The selected articles included information from 402 patients, where the information on the type of study, intervention, or technique performed, and the results obtained was found (Table 2).

An analysis of the content of studies included in this review, according to the variables included, is presented below.

### 3.1. Type of Study

Overall, five randomized controlled trials (9,21–24), two pilot studies (21,27) and four descriptive studies [25,28,29,30] were included.

### 3.2. Application of the Jadad Scale

Table 3 analyzes the methodological quality of the clinical trials included in this review. Most articles did not indicate the method used to generate the sequence of randomization, whether adequate blinding was performed, or the description of losses, abandonments, and patient follow-up.

Only one trial was of low quality according to the JADAD scale [20] (Table 3). All the studies asserted that they used a randomization method. Three studies got less score due to the lack of information on report losses and exclusions [22,23] or if the blinding of the evaluator and the participants was carried out according to the allocated group [9,22].

According to the results of the different studies, it is possible to state the reliability of the different methods of intervention assessed. Among those with better evidence, EMST may be a restorative and reliable treatment for dysphagia in those with PD [21,22,29]. In this same category would fall the video-assisted swallowing therapy (VAST), which proved reliable for patients with Parkinson’s disease [23]. On the other hand, the effects of neuromuscular electrical stimulation (NMES) as adjunct to therapy on the quality of life in patients with Parkinson’s disease and oropharyngeal dysphagia are found inconclusive [9,24,27]. This would also be the case for the effortful swallow maneuver reinforced by using biofeedback, the effects of bolus consistency, TTS, or motor swallowing exercises, as they all appear to be inconclusive therapeutic resources that need further evaluation [25,26,28,30].

### 3.3. Treatment/Intervention or Rehabilitation Techniques

Felix et al. [26] proposed the implementation of the swallow maneuver with effort (with biofeedback), a rehabilitation program that included eight maneuvers to facilitate swallowing. Swallowing with the exercise maneuver consisted of asking the patient “to swallow by contracting the muscles of the mouth and throat with the greatest possible force.”

Byeon [22] proposed a simultaneous treatment of postural techniques and expiratory muscular strength training in the improvement of swallowing function in patients with PD, since according to the author, improvements were observed when both techniques were applied at the same time (vitalization of the suprahyoid muscle, reduction of aspiration and opening of the upper esophageal sphincter). Regan et al. [30] demonstrated that tactile thermal stimulation (TTS) accelerates involuntary pharyngeal swallow.

Authors such as Troche, Byeon or Pitts et al. [21,22,29] proposed an EMST device to improve voluntary coughing, increasing the acceleration of cough volume. Troche et al. [21] promoted a restorative treatment (EMST) for dysphagia in PD in which the experimental group improved hyolaryngeal function and swallow safety compared to the sham group, with Class I evidence. Previously, Troche et al. (2008) suggested that thicker consistencies should yield a safer swallow in those with PD [28].

Argolo et al. [25] established oral motor exercises (10 repetitions of sustained vowel phonation of /a/, pushing of plosive phonemes in a forceful manner; sucking of wet gauze; tongue-hold swallowing; modified supraglottic maneuver (emission of /a/ vowel in a forceful manner) before breathing again. And five repetitions of ascending and descending gliding phonations of the vowels /a/ and /u/; and three series of five repetitions of tongue rotation, each side, in the oral vestibule. It positively impacted the quality of life and swallowing complaints.

Manor et al. [23] used video-assisted swallowing therapy (swallowing exercises and compensatory therapy technique carried out with different consistencies of food and liquid), which significantly decreased the presence of residues in the pharynx, and therefore improved swallowing. Speech therapy for dysphagia, in Baijens’s study, included rehabilitation techniques to improve or restore the swallowing physiology and to facilitate oral dietary intake, such as exercises to improve sensorimotor integration and muscle strength, as well as (postural) maneuvers [24].

Heijnen [9] made a comparative study of neuromuscular electrical stimulation at sensor or motor level and traditional therapy regarding the effect on the quality of life, but the differences between the groups were nonsignificant. Baijens et al. [27] attempted to demonstrate the effect of a surface electrical stimulation (SES) session using different electrode positions on PD patients with dysphagia, but the results were the same for both healthy control subjects and PD patients. The results could be attributed to the placebo effect in control subjects. These three studies [9,24,27] explored the effects of SES of the neck in patients with PD. The studies evaluated the traditional treatment of physiotherapy that consisted of maneuvers for the respiratory tract, postural compensation, modification of the bolus, and motor exercises together with SES. The results were not significant for SES but were significant when traditional physiotherapy techniques were applied [24].

Regarding the evaluation of dysphagia, different scales and measures were used in the articles: O’Neil Dysphagia Outcome and Severity Scale (DOSS) [26,30], a visual analogue scale, the Dysphagia Severity Scale (DSS) [9], and the Functional Dysphagia Scale, based on videofluoroscopic studies (VFS) [24].

Many studies used a Standardized Videofluoroscopic Swallowing (VFS) protocol [9,21,22,23,24,25,26,27,28,29,30], or fiber-optic endoscopic evaluation of swallowing (FEES) [9,23,24].

Swallow timing was assessed using a Digital Swallowing Workstation [28] or the oral and pharyngeal transit times were measured in milliseconds with the Avidemux software [25]. The questionnaire on swallowing disturbances evaluated the presence of swallowing problems [23].

The Penetration/Aspiration scale (P/A scale) was used to indicate the safest swallow [21,27,28,29]. Expiratory Muscle Strength Training (EMST) [25,29] was employed to measure the parameters of voluntary coughing.

The level of disability was measured through the Hoehn and Yahr scale in all studies [9,21,22,23,24,25,26,27,28,29,30]. Motor function or severity was assessed using the Unified Parkinson’s Disease Rating Scale (UPDRS) [21]. The patient’s degree of enjoyment from food was assessed with the pleasure of eating scale [23]. Functional Oral Intake Scale (FOIS) [9] was used to evaluate the diet.

The quality of life was measured using the following scales: the Swallowing Quality of Life Questionnaire (SWAL-QOL) [9,21,23,25], MD Anderson Dysphagia Inventory [MDADI] [9] and the Swallowing Quality of Care (SWA-CARE) [23]. Cognition was evaluated using the Mini Mental State Examination (MMSE) [9,21,22,23,24,25,28].

## 4. Discussion

PD is becoming more frequent as the global human population is aging [3,4,26]. In PD, dysphagia is very common with serious and negative consequences for physical health and quality of life [31,32,33]. Despite this, there is not enough evidence to determine the quality of interventions being performed to treat dysphagia during PD [9,23,25,31]. Today, the most common treatment focuses on changing the texture of the diet, although there are other techniques aimed at improving the quality of life of the patients suffering from dysphagia [21,28]. Different techniques have been proposed by nurses, speech therapists, physiotherapists, occupational therapists, and doctors [9,21,22,23,24,25,26,27,28,29,30].

This systematic review identifies the rationing instruments and interventions in each trial. Several rehabilitative therapies as EMST or NMES have been successful in swallowing and reducing the risk of choking, aspiration or improving oropharyngeal function [9,21,22,29]. An improvement was seen in the degenerative function (coordination, speed, and volume), quality of life, and social relationships of people with PD [23,25]. However, some experimental intervention studies included a small or very small number of patients. Only 4 of 11 studies included more than 40 participants; therefore, the extent of results is also limited [9,21,23,24].

Most of the articles showed an improvement in the degenerative function after applying the described techniques and, thus, the improvement in the quality of life and relationship of these patients with the environment was notable [21,22,23,25,26]. However, SES did not show any positive influence in traditional speech therapy [24]. The frequency and duration need further investigation to construct a clinical decision-making model for the treatment options in this patient population.

Previously, another systematic review [34], based on the literature and experience of its research team, established a therapeutic strategy to improve PD symptoms such as dysphagia, posture, step, bradykinesia, depression, tremor, and loss of strength. Van Hooren, Baijens, Voskuilen et al. [11], in a systematic review where 12 articles were used, described different techniques as oral motor exercises, EMST, video-assisted swallowing therapy, surface electrical stimulation, thermal stimulation, touch, chewing gum effect. Another study [31] reviewed the literature on compensatory and rehabilitation interventions for dysphagia in PD and argued that rehabilitation methods could possibly have a greater potential to increase the safety of swallowing and, thus, to improve the quality of life. However, compensatory methods for the treatment of dysphagia have received more attention than rehabilitation methods, and yet, none of these have a strong evidence base.

Another article [35] showed that in the first three months after deep brain stimulation of the subthalamic nucleus, rehabilitation was required in a small proportion of patients with PD. This study supported the idea that a fast-track rehabilitation program, similar to that of Jiang et al. [36], may be useful but yet it would not be necessary for the entire surgical patient population. However, Gadenz’s systematic review described the benefits of the repetitive transcranial magnetic stimulation (noninvasive transcortical stimulation) and its applicability in these neurogenic disorders related to communication and deglutition, particularly from the studies on stroke patients, but this finding is still uncertain [37]. He described that some studies explored the stimulation of the dorsolateral prefrontal cortex because it could improve clinical symptoms, including speech-language impairment in PD [37,38].

This review has evaluated 11 studies that assessed the effects of different therapies for swallowing disorders due to PD, and all of them used a standardized videofluoroscopic swallowing protocol for an objective evaluation [9,21,22,23,24,25,26,27,28,29,30].

The data collected in the anamnesis, clinical, and instrumental evaluation of PD patients should be detailed to provide an understanding of the disorder and its diagnosis and promote treatment planning and treatment evolution [9,11,21,22,23,24,25,26,27,28,29,30]. Some of the scales and measures that had been used to evaluate these patients with dysphagia were the Dysphagia severity scale, the oral and pharyngeal transit time, the swallowing disturbances questionnaire, the penetration/aspiration scale, expiratory muscle strength, the unified Parkinson’s disease rating scale, the pleasure of eating scale, the functional oral intake scale, the swallowing quality of life questionnaire, the MD Anderson Dysphagia Inventory, and the Swallowing quality of care questionnaire [9,21,22,23,24,25,26,27,28,29,30].

The risk of bias, as evaluated by the JADAD scale [20] in the clinical trials, indicated that most studies had good methodological quality, although some of them did not report the blinding method or describe losses and exclusions. Further research is needed to expand the knowledge about new treatments and techniques in PD.

### Limitations and Future Perspectives

The limitations of this review article are directly related to the limitations of the selected articles. Most articles used a small sample, so the results are not sufficiently reliable, and thus the authors propose that larger samples should be recruited for future studies [22,25,26,27,28,30]. Another limitation is the patients’ knowledge of the applied therapy, so in many cases, the effect could have been due to the placebo effect and not to the technique itself.

In order to mitigate some of the effects or limitations of this review, we employed comprehensive search strategies to avoid biases in the information gathering process, making a critical assessment and synthesis of the studies. In addition, the data obtained were extremely heterogeneous because of the differences between the designs and therapies or rehabilitation interventions applied in the reviewed studies.

As research on this topic becomes more rigorous, further revisions will be required on interventions that have changed or improved over time. As dysphagia rehabilitation is a central aspect of the working of a speech-language therapist in many countries, this term “speech-language therapy” could be considered in future reviews and more articles can be retrieved in the search.

There is insufficient scientific evidence to compare the effectiveness of the various techniques described, and well-designed studies are needed to determine the value of the exercises used in the various fields of application, with appropriate follow-up. Most of the studies that we reviewed state that physiotherapeutic treatment for dysphagia in PD accelerates swallowing improvements.

We would like to highlight the importance of well-designed experimental research that overcomes the limitations observed and obtains more reliable results on the treatment of dysphagia, as dysphagia is a serious and vital sequela of PD. A multidisciplinary team should be informed and prepared to offer a correct intervention in PD. Once the investigations are more rigorous on the subject, further revisions will be required.

More work needs to be done to establish or define what type of therapies, rehabilitation techniques, exercises, or maneuvers are the most effective in dysphagia and to develop agreed protocols. These interventions need to be evaluated with regard to intensity, frequency, duration, consistency, side effects, institution versus home based, individualized versus standardized training, practicability in real life, and quality standards [12]. It is essential to optimize the quality of PD care and minimize its cost, in order to make it efficient and equitable where it is provided [18].

## 5. Conclusions

This review compiled several techniques and treatments used for swallowing problems in PD patients, such as compensation strategies, swallow maneuvering, expiratory muscle strength training, together with postural treatment, traditional physiotherapy techniques, muscular training of the tongue, pharynx, larynx, and respiratory apparatus, and surface and neuromuscular electrical stimulation. Most of the results obtained with the application of these techniques described in the selected articles support an improvement in the degenerative function, even though these results are not of high quality in most of the studies. Further investigations concerning the clinical applicability of these therapies based on well-designed randomized controlled trials are needed with larger patient populations. This will give a correct estimate of the effectiveness, long-term effects, and the possibility of new techniques.

## Figures and Tables

**Table 1:** Systematic syntax in the databases.

Search in the Databases	Results
**SCOPUS**	
(Parkinson disease) AND (deglutition disorders) AND (rehabilitation)	33
(Parkinson’s disease) OR (Parkinson and disorders) AND (deglutition disorders) OR (dysphagia) AND (physical therapy specialty) or (physiotherapy) OR (physiotherapy modalities)	35
**Medline**	
Disease of Parkinson AND dysphagia AND rehabilitation	37
Disease of Parkinson AND deglutition disorders AND rehabilitation	28
(Parkinson disorders OR Parkinson’s disease) AND (dysphagia OR oral care OR deglutition disorders) AND (physiotherapy OR physical therapy) AND treatment	25
**Elsevier**	
Parkinson AND disfagia AND fisioterapia	47
**Pubmed**	
((Parkinson disorders OR Parkinson disease)) AND (dysphagia OR deglutition disorders)) AND ((physiotherapy OR physical therapy modalities)) AND mouth rehabilitation	28

**Table 2:** Study characteristics and main results.

Authors, Year	Type of Study and Participants	Description of Intervention and Duration. Results
Heijnen et al., 2012 [9]	Randomized Trial. 109 patients. 3 groups (30 patients). NMES vs. Traditional Therapy.	Intervention: Group 1: oral motor exercises, airway protection maneuvers and postural compensation. Group 2 and 3: previous exercises as group 1 plus NMES of the suprahyoid muscle (frequency 80 Hz, pulse width 700 µs). Group 2 received stimulation at the motor level and Group 3 at sensory level. Duration: 13–15 sessions of half an hour each, for five consecutive days per week (3–5 weeks). Evaluation after 3 months of treatment. Results: All groups showed significant effects on the severity scale of dysphagia (Group 1, median = 59, *p* < 0.001; Group 2, median = 72, *p* < 0.001; Group 3, median = 74, *p* = 0.005), as well as improvements in SWAL-QOL (*p* = 0.001) and MDADI (Global assessment *p* < 0.001). Minor changes were recorded among the groups. A more comprehensive study may be necessary to clarify these preliminary findings.
Troche et al., 2010 [21]	Randomized trial. 60 Parkinson patients. EG = 30 CG = 30	Intervention: The 30 participants of EG completed EMST. It was a device through which air flows for the expiratory muscles. Duration: 4 weeks, 5 days/week, 20 min/day. For the CG, the device provided little or no load. Results: There were no differences between the pre-treatment groups (*p* = 0.881). The EMST group (EG) demonstrated an improvement in oropharyngeal function during swallowing (0.61; 95% CI 0.10 to 1.11), these findings were not evident for the CG.
Byeon, 2016 [22]	Clinical trial. 33 patients. Group A: 18 only EMST Group B: 15 “postural techniques plus EMST”	Intervention: EMST: the nose was blocked with clips and later, with a mouthpiece, the patients were asked to perform different exercises (inspiration and expiration fast and maximum). Applied for 20 min/day, 5 days/ week. Postural techniques: tilting of the chin, rotation of the head, tilting of the head, bringing the head to extension and flexion, for 30 min/ session and 5 days/week. Duration: 4 weeks. Results: the simultaneous performance of postural techniques and EMST is more effective than using only EMST in dysphagia (22.5 vs. 16.2; *p* < 0.05). These postural techniques are effective for the swallowing rehabilitation of PD patients who have limited movements of the tongue base or weakened laryngeal raise.
Manor et al., 2013 [23]	A randomized study. 42 patients. GE = 21 GC = 21	Interventions: swallowing exercises and compensatory therapy technique. The only difference was the implementation of the video-assisted tool during each therapy session, for education and assistance in GE. Evaluation at baseline, at 4 weeks and 6 months. Duration: Both groups underwent 6 interventional sessions of 30 min. Results: Both conventional swallowing therapy and VAST approaches proved effectiveness (14.27 vs. 14.65; *p* = 0.25). The VAST highlights the connection between patients’ level of quality of life, degree of pleasure of eating and the level of satisfaction with the therapy.
Baijens et al., 2013 [24]	Randomized controlled trial. *N* = 90 patients. Three groups.	Intervention: The three groups received traditional speech therapy: diverse airway protecting maneuvers; postural compensation maneuvers; bolus modification and oral intake of different sort of food; swallowing saliva; and oral motor exercises. In addition, two groups received SES, either motor- or sensory-level stimulation. Duration: Daily 30 min, 15 days. Results: No statistically significant differences in FEES (2.67; 95% CI [1.65,4.33]) and VFS (11.30; 95% CI [6.47,19.71] outcome variables were found between any of the three treatment groups (no significant). A therapeutic effect of traditional therapy is suggested, without any additional influence of SES. Further research is required.
Argolo et al., 2013 [25]	A before–after trial. 15 consecutive patients	Intervention: Motor swallowing exercises (increase of the strength and range of motion of the mouth, larynx and pharyngeal structures, coordination between breathing and swallowing, and airway protection). Duration: Twice a day, 5 days/week, 5 weeks. Results: Motor swallowing exercises may reduce swallowing disorders in PD patients without lingual pumping and dental absence (beta standardized coefficient = −16.6, 26.2; *p* = 0.02, 0.002, respectively). Reduction in swallowing disorders was not related with QOL improvement (0.13, [95% CI, 0.6–0.4], *p* = 0.63).
Felix et al., 2008 [26]	A pilot study 10 patients. EG: 4 patients CG: 6 patients	Intervention: Swallow maneuver with effort and biofeedback. Each session included 8 maneuvers: 4 involved saliva and the other 4 solids (cookies). This effort maneuver was induced by requesting “swallow contracting the muscles of the mouth and throat with the greatest possible force”. In addition, a biofeedback device was adapted. Duration: daily sessions (2 weeks) during which a biofeedback resource was used. Results: Patients who had problems with water intake did not show such a problem after rehabilitation, unlike the CG. There was a numerical similarity of the results obtained with the swallowing of saliva or of biscuits (variance = 4.41). None of the patients exhibited difficulty in coughing, drowning or voice disturbance as a result of swallowing after the rehabilitation program (*p* < 0.001). The effortful swallow maneuver reinforced by using biofeedback was a therapeutic resource. An additional assessment with a larger number of patients is warranted.
Baijens et al., 2012 [27]	A pilot study Idiopathic Parkinson’s disease *n* = 10 Healthy control subjects *n* = 10	Intervention: a session of SES using three different electrode positions during videofluoroscopy of swallowing. Temporal, spatial, and visuoperceptual variables were scored. Duration: a single session. Results: showed that SES could alter swallowing in Parkinson’s disease. However, these effects may have been caused mainly by the placebo effect. The changes measured from SES were found in both healthy control subjects and Parkinson patients and the direction of change would not likely benefit swallowing (*p* > 0.05). Laryngeal vestibule duration (seconds), Position I versus II: 0.03 (−0.013, 0.07) N.S.; Position III versus I: 0.022 (−0.025, 0.069) N.S. Duration horizontal hyoid motion (seconds), Position II versus III: 0.488 (−0.139, 1.116) N.S.; Position III versus I: 0.248 (−0.379, 0.876) N.S.
Troche et al., 2008 [28]	Prospective cohort study. 10 patients	Intervention: The study quantified the effects of bolus consistency on specific aspects of movement and bolus flow abnormalities (thin and thick bolus), including swallowing time and number of tongue pumps. Duration: six thin and six pudding-thick boluses were analyzed. Results: a significant difference in oral transit time as a function of bolus thickness, with increased oral transit time with thicker boluses (6.70 vs. 14.30; *p* = 0.004). No significant difference in pharyngeal transit time between the two consistencies was found (10.35 vs. 10.65; *p* = 0.910).
Pitts et al., 2009 [29]	Prospective cohort study. 10 patients	Intervention: EMST was applied to improve cough and swallow function at home. Duration: 4 weeks, 5 days/week, 5 sets of 5 breaths a day. Results: EMST was an effective treatment for PD patients with risk of aspiration. There was a significant decrease in the compression phase duration (Z = 2.803; *p* = 0.005) and expiratory phase rise time (Z = 2.492; *p* = 0.01) (which resulted in a significant increase in cough volume acceleration (Z = 2.497; *p* = 0.01)).
Regan et al., 2010 [30]	A cohort study 13 patients	Intervention: TTS requires little clinical training and has few known contraindications. A Thermo-Stim^TM^ (Luminaud, Mentor, OH) implement was employed. Liquid barium and barium paste were the two chosen consistencies. Duration: measures before and after TTS. Results: TTS significantly reduced pharyngeal transit time (0.20 s; *p* = 0.004), total transit time (0.48 s; *p* = 0.049), and pharyngeal delay time during swallowing (0.20 s; *p* = 0.002).

PD = Parkinson Disease; EG = Experimental group; CG = Control group; N.S. = No significant; EMST = expiratory muscle strength training; NMES = Neuromuscular Electrical Stimulation; TTS = Thermal–tactile stimulation; SES = surface electrical stimulation; SWAL-QOL = Swallowing Quality of Life Questionnaire; MDADI = MD Anderson Dysphagia Inventory; VAST = Video-assisted swallowing therapy; FEES = fiber-optic endoscopic evaluation of swallowing; VFS = Videofluoroscopic Swallowing.

**Table 3:** Risk of bias. Application of the Jadad scale to the clinical trials of this review.

Article	The Study Was Described as Randomized	The Method Used to Generate the Sequence of Randomization and Its Appropriation	Study Described as Double-Blind	The Method of Blinding Is Described and Appropriate	There is a Description of Follow-Up and Drop-Out Losses	TOTAL <3/5 Low Quality
Heijnen et al., 2012 [9]	YES	YES	NO	NO	YES	3/5
Troche et al., 2010 [21]	YES	YES	YES	YES	YES	5/5
Byeon, 2016 [22]	YES	YES	NO	NO	NO	2/5
Manor et al., 2013 [23]	YES	YES	YES	YES	NO	4/5
Baijens et al., 2013 [24]	YES	YES	YES	YES	YES	5/5
